# Systems for electronic documentation and sharing of advance care planning preferences: a scoping review

**DOI:** 10.1080/09699260.2024.2339106

**Published:** 2024-04-26

**Authors:** Hüsna Sarıca Çevik, Catharina Muente, Felix Muehlensiepen, Jacqueline Birtwistle, Alexander Pachanov, Dawid Pieper, Matthew J. Allsop

**Affiliations:** a Leeds Institute of Health Sciences, School of Medicine, Faculty of Medicine and Health, University of Leeds, Leeds, UK; b Department of Family Medicine, Ankara University Faculty of Medicine, Ankara, Turkey; c Faculty of Health Sciences Brandenburg, Brandenburg Medical School (Theodor Fontane), Institute for Health Services and Health System Research, Rüdersdorf, Germany; d Center for Health Services Research, Brandenburg Medical School (Theodor Fontane), Rüdersdorf, Germany; e Evidence Based Practice in Brandenburg: A JBI Affiliated Group, University of Adelaide, Adelaide, Australia

**Keywords:** Advance care planning, digital technology, Resuscitation Orders, Resuscitation Decisions, Electronic Health Records, Health Information Exchange

## Abstract

Digital approaches to support advance care planning (ACP) documentation and sharing are increasingly being used, with a lack of research to characterise their design, content, and use. This study aimed to characterise how digital approaches are being used to support ACP documentation and sharing internationally. A scoping review was performed in accordance with the JBI (formerly Joanna Briggs Institute) guidelines and the PRISMA 2020 checklist, prospectively registered on Open Science Framework (https://osf.io/xnrg3). MEDLINE, EMBASE, PsycINFO, ACM Digital, IEEE Xplore and CINAHL were searched in February 2023. Only publications in English, published from 2008 onwards were considered. Eligibility criteria included a focus on ACP and electronic systems. Out of 2,393 records, 34 reports were included, predominantly from the USA (76.5%). ACP documentation is typically stored in electronic health records (EHRs) (67.6%), with a third (32.4%) enabling limited patient access. Non-standard approaches (*n* = 15;44.1%) were the commonest study design of included reports, with outcome measures focusing on the influence of systems on the documentation (i.e. creation, quantity, quality, frequency or timing) of ACP information (*n* = 23;67.6%). Digital approaches to support ACP are being implemented and researched internationally with an evidence base dominated by non-standard study designs. Future research is needed to extend outcome measurement to consider aspects of care quality and explore whether the content of existing systems aligns with aspects of care that are valued by patients.

## Introduction

Palliative care aims to alleviate suffering for people living with progressive, life-limiting illnesses and improve quality of life through a holistic, person-centred, and multidisciplinary approach [1]. A means of facilitating person-centeredness in the delivery of care is through advance care planning (ACP). ACP can be characterised as a process that helps people understand and express their personal values, life goals and preferences about their future medical treatment. This process is connected to the ambition of ensuring that people with serious and chronic illnesses receive medical care that is consistent with their values, wishes, and preferences [2]. It is typically an ongoing, iterative process involving the elicitation of a patient’s goal and values for care often involving their families and healthcare providers [3]. By determining goals, values, wishes, and preferences for care in advance, it is intended that there is concordance in the care and treatments that are subsequently received [2]. There is evidence that ACP helps to ensure that end-of-life care wishes are known to healthcare providers and are more likely to be followed [4]. In addition, family members can benefit from documentation of end-of-life care preferences, including improved outcomes relating to stress, anxiety, and depression [4]. A lack of documented ACP can, in contrast, lead to medical interventions that are not in line with patients’ wishes [5].

To enable the implementation of documented care preferences, it is crucial to make them accessible to healthcare providers so that they can readily and easily access ACP information when necessary. This accessibility can be achieved, for example, through digital approaches such as storing ACP documentation in an electronic health record (EHR). Across multiple countries, there has been an evolution of digital approaches to support the collection, documentation, and sharing of ACP information between healthcare services and settings [6]. Internationally, this approach has been developing in countries including the USA [7, 8], Australia [9], and England [6]. Whilst there have been multiple examples cited for digital approaches, it is unclear how these have been adapted for different health systems, which information is collected and shared, and how data sharing is coordinated across multidisciplinary teams involved in the delivery of palliative and end-of-life care.

This Scoping Review aims to characterise how ACP documentation is currently integrated into electronic systems. We consider a scoping review to be an appropriate procedure, as heterogeneous studies and findings can be assumed due to the diverse approaches to storing ACP documentation in different countries and their respective healthcare systems.

Three research questions (RQs) are addressed:

- RQ 1: What are the characteristics and structure of systems being used to support electronic documentation and sharing of ACP preferences?
- RQ 2: What is the focus and type of existing evidence on the use of electronic documentation and sharing of ACP preferences?
- RQ3: How are systems for electronic documentation and sharing of ACP preferences being evaluated?

## Methods

This project was a collaborative project between the University of Leeds, UK, and the Brandenburg Medical School, Germany. A team approach was adopted in the conduct of the scoping review. The team met regularly throughout the review process, including data extraction, analysis and presentation [10]. This scoping review followed the updated JBI (formerly Joanna Briggs Institute) guide for scoping reviews [10]. In addition, the Preferred Reporting Items for Systematic Reviews and Meta-Analyses extension for Scoping Reviews (PRISMA-ScR) checklist was used [11].

### Protocol and registration

This scoping review is registered on Open Science Framework (https://osf.io/xnrg3). An amendment was made to the inclusion criteria, which is detailed below.

### Eligibility criteria

#### Inclusion criteria

- Focus on advance care planning
- Digital health technologies
- Articles in English
- Articles available in full-text
- Articles published from 2008 onwards

#### Exclusion criteria

- No full text available
- Other languages than English

We only included studies published from 2008 onwards, as ACP supported by digital health technologies has only been developing in England since 2008 [12]. To our knowledge, England is the first country to address this topic. An inclusion criterion, removed between protocol and undertaking the review, was ‘People with chronic progressive illness’. We sought to adopt a broader focus on evidence that can inform digital advance care planning approaches, not confining the inclusion of studies limited to people with chronic, progressive illnesses.

### Information sources

We searched the following seven bibliographic databases: MEDLINE, EMBASE, PsycINFO, ACM Digital, IEEE Xplore and CINAHL. Grey literature was searched on greylit.org and government websites. We also searched Google Scholar and we manually searched for additional studies by cross-checking the reference lists of all included studies. In addition, backward citation screening was performed for all included studies. For the conference abstracts we found, we tried to obtain the full text through additional searches or by contacting the study authors. We contacted one author and did not receive a response, but a full-text version was subsequently obtained. Reminders were not sent.

### Search strategy

The search strategy was developed by the research team in collaboration with a librarian. It is based on the Peer Review of Electronic Search Strategies (PRESS) guideline [13]. The search strategy includes general terminology and names of specific systems that we are aware of or that have been referenced in a previous review [14]. For all databases, the literature search was conducted in February 2023. However, the grey literature was searched in June 2023. The search strategy is presented in Appendix 1.

### Source of evidence selection

**Figure 1.**
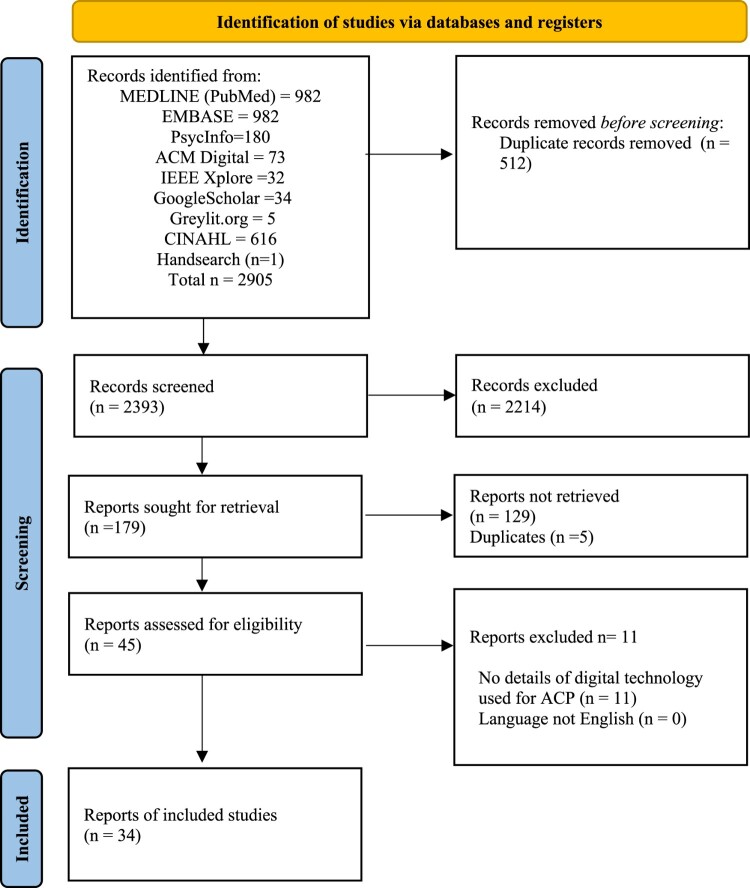
PRISMA flowchart for identification, screening, and inclusion of studies.

After compiling the search results, all citations were imported into the bibliographic manager, EndNote. All duplicates were removed by using an automated function in EndNote [15]. Subsequently, title and abstract screening was conducted by two independent reviewers (HSC and FM) against our defined eligibility criteria, via Rayyan – a web and mobile application for systematic reviews [15]. Any disagreements were solved by consensus or by the decision of a third reviewer (MA). Full texts were obtained for references that were deemed potentially relevant. Each full text was screened independently by two reviewers from a group of three reviewers in total (HSC, CM, MA) using our eligibility criteria. The reasons for the exclusion of full texts were documented and entered in the flow chart, provided in Figure 1. In all steps, disagreements were resolved by discussion and consensus between the two reviewers or by involving a third reviewer in case of doubt. The list of included studies is provided in Table 1.

**Table 1.** Summary of included studies.

Author(s)	Year	Country	Study design	Population and sample size (*n*)	Description of system(s) involved	Name of system being described
Auret et al.	2019	Australia	Quantitative (observational)	*N* = 73 (Physicians (*n* = 26), nurses (*n* = 15) other HCPs (*n *= 12), audit of patients (*n* = 20))	EHR	NA
Brungardt et al.	2019	USA	Quantitative (observational)	*N* = 105 (patients)	EHR	My Health Connection
Fin et al.	2016	USA	Quantitative (observational)	*N* = 900 (people who were users of the MyDirectives platform)	Standalone system not involving an EHR	MyDirectives
Goodwin et al.	2021	New Zealand	Other	*N* = 3,238 (The findings were obtained from an analysis of a database of 3238 people who had finalised an AC Plan between December 2013 and December 2019.)	EHR	eAC Plans
Hall et al.	2012	UK (Scotland)	Qualitative	*N* = 22 (purposive sampling of practice nurses (3 interviews), GPs (12 interviews), a practice manager (1 interview) from practices using different computing software systems and patients and/or carers (6 interviews for whom an ePCS had been completed))	EHR	Electronic Palliative Care Summaries (ePCSs)
Halpert et al.	2021	USA	Quantitative (observational)	*N* = 426 (patients aged 75 years or older who had an upcoming appointment with their provider within 2 weeks and who had no documentation of ACP in their chart)	EHR	Epic EMR, which includes an ACP activity tab
Harrington et al.	2020	UK (England)	Mixed	*N *= 159 (data on gerontology inpatients (*n* = 133) with electronic do not attempt cardiopulmonary resuscitation (e-DNACPR) decisions and survey data from staff (*n* = 26) including senior and junior doctors, and medical trainees)	EHR	NA
Holt et al.	2019	USA	Other	NA	Standalone system not involving an EHR	Oregon’s online registry of out-of-hospital DNR orders
Jordan et al.	2019	USA	Qualitative	*N* = 46 (patients, mean age: 49, 63% female)	EHR	My Health Connection
Klugman et al.	2013	USA	Quantitative (observational)	*N* = 371 (platform users)	Standalone system not involving an EHR	TexasLivingWill.org (TLW), NVLivingWill.com (NVLW)
McDarby et al.	2021	USA	Other	NA	Other	ACP Tools (iOS and Android) BIDMC Health Care Contingency Plan – Personal MedStar CR My Dot Mediq My Directives My Health Proxy My Living Will Paper Health
Millington-Sanders et al.	2012	UK	Quantitative (observational)	*N* = 597 (patients)	Standalone system not involving an EHR	Coordinate My Care (CMC)
Mills et al.	2021	Australia	Mixed	*N* = 170 (Medical, nursing, and allied health practitioners working in palliative care)	Standalone system not involving an EHR	MyHealth Record
Moses et al.	2020	USA	Other	NA	EHR	NA
Nakagawa et al.	2014	USA	Quantitative (observational)	*N* = 93 (patients)	EHR	NA
Neubauer et al.	2015	USA	Quantitative (observational)	*N* = 5,467 (patients)	EHR	My Choices, My Wishes
Obel et al.	2014	USA	Quantitative (observational)	*N* = 48 (patients)	EHR	NA
Portz et al.	2020	USA	Quantitative (observational)	*N* = 3292 (UCHealth patients who interacted with the My Health Connection patient portal, advance care planning tools, specifically by completing an electronic MDPOA form or sending an electronic message to the advance care planning support team)	EHR	My Health Connection
Portz et al.	2020	USA	Qualitative	*N* = 24 (participants from the KPCO EMR with inclusion criteria included: ≥65 years of age, KPCO member for ≥ 1 year, presence of multiple chronic conditions **(**Charlson Comorbidity Index > 2), and a patient with one of the participating KPCO study clinics)	EHR	My Health Manager (Kaiser Permanente Colorado’s (KPCO) patient portal)
Reidy et al.	2017	USA	Mixed	*N* = 27 (hospitalists, residents, and nurse practitioners on conducting goals-of-care conversations – although other survey work also outlined)	EHR	Luminat
Riley et al.	2013	UK (England)	Other	NA	Standalone system not involving an EHR	Coordinate My Care
Rolnick et al.	2021	USA	Quantitative (experimental)	*N* = 91 (Adults with gastrointestinal and lung malignancies were recruited from the Penn Medicine Infusion Center. 46 randomly allocated to the web AD and 45 to paper)	EHR Plus	Our Care Wishes
Saiki et al.	2017	USA	Other	NA	EHR	Goals of Care template, as part of TEAM approach (Time, Education, Assessment, and Management)
Seecof et al.	2022	USA	Other	*N* = 50 (Patients at the Center for Healthy Aging aged 65 or older without documented ACP in the EMR)	EHR	NA
Serrano-Eanelli et al.	2019	USA	Other	*N* = 331 (medical records of patients)	EHR Plus	New York State eMOLST (electronic Medical Order for Life-Sustaining Treatment form)
Seuli Bose-Brill et al.	2016	USA	Quantitative (experimental)	*N* = 50 (patients)	EHR Plus	MyChart
Shervin Esfahani et al.	2020	USA	Other	*N* = 365 (Retrospective record review of all ICU deaths across the system in 1 year)	EHR	SmartPhrase (a tool in the Epic EMR system)
Stepan et al.	2019	USA	Other	*N* = ∼15,600 (patient records of all new patients with a diagnosis of advanced or metastatic cancer who had a documented advance directive and/or ACP conversations with an ambulatory care provider (physicians, fellows, residents, physician assistants, nurse practitioners, nurses, social work counsellors, and chaplains) by their third office visit)	EHR	NA
Tieu et al.	2017	USA	Quantitative (observational)	*N* = 200 (patients within the Division of Primary Care Internal Medicine, aged 65 and older, with access to the secure PEM system and without an AD on file, were included for randomization)	EHR Plus	Mayo Clinic Patient Online Services system
Walker et al.	2018	USA	Quantitative (observational)	*N* = 414 (primary care patients at the San Francisco Veterans Affairs Medical Center, were $60 years old, and had $2 chronic/serious health conditions)	EHR	Prepare for your care (www.prepareforyourcare.org)
Wilson et al.	2020	USA	Other	NA	EHR	Epic Systems EMR
Wu et al.	2022	USA	Quantitative (observational)	*N* = 187,690 (unique patients 65 years and older with at least one clinical encounter at an SHC outpatient clinic over the study period)	EHR	Epic Systems
Wye et al.	2016	UK	Mixed	*N* = 3,594 (Data from 1,022 North Somerset and 2,572 Somerset palliative patients; interview participants = 101 professionals, 49 by telephone, 29 face to face and 23 informally)	EHR	Adastra
Zive et al.	2016	USA	Other	NA. Intended for those nearing end of life.	EHR	ePOLST

Key: EHR = Electronic Health Record; EMR = Electronic Medical Record; NA = Not applicable.

### Data extraction

The standardised data extraction form offered by JBI [16] was used as a basis and adapted to the research questions of this scoping review. The spreadsheet software Microsoft Excel was used for this purpose. A pilot test of the data extraction form was conducted on a sample of five publications by three reviewers (MA, HSC, CM) independently to assess completeness and applicability. The results of the pilot testing were discussed with the whole review team and necessary changes were made to the extraction form. Disagreements were resolved through discussion. Data extraction was completed by team members (MA, HSC, CM) with all extracted data checked by a second reviewer (MA, HSC, CM) to ensure accuracy and completeness.

### Data items

Data extraction included the following elements:

- Authors
- Title
- Year
- Country (in the first author’s affiliation)
- Aim
- Study type
- Methodology
- Publication type
- Population and sample size
- Description of system(s) involved (i.e. EHR system, EHR and other system(s), or standalone system not involving an EHR)
- Name / title of the system being described
- Settings (care or universal access) involved in introducing the system
- Description of the ways that different healthcare providers interact with the system and responsibilities of providers across different care settings when interacting with the system
- Processes for interacting with the system (healthcare provider and/or patient) or receiving communication from the system (e.g. healthcare provider access via EHR template, patient access via patient portal)
- Content of the system (i.e. details of types of data recorded by system)
- Outcomes evaluated (study outcomes)
- Authors’ conclusion
- Source of funding

### Study risk of bias assessment

As this is a scoping review, no risk of bias assessment was performed. Critical appraisal or risk of bias assessment is generally not recommended for scoping reviews. Rather, the aim is to capture the available evidence [17].

### Synthesis of results

The results were synthesised using qualitative content analysis as suggested by the JBI Scoping Review Methodology Group [18]. This included some form of categorisation to simplify the findings for the reader, particularly through the use of thematic networks [19]. CM has developed a first draft of the code system which was developed through discussion with the research team. Appendix 2 includes the definitions used to guide the categorisation of extracted data. The software MAXQDA® Software (Verbi Software Ltd, Berlin, Germany), version 2020, was used for the data analysis. The codes developed were discussed and adapted in regular meetings of MA, HSC and CM. A quality appraisal of the included studies was not performed.

### Stakeholder involvement

This Scoping Review was developed with the support of patient organisations in the UK that specialise in the development and dissemination of patient-centred material to support decision-making for people with life-limiting or life-threatening conditions. The organisations contributed to the focus of the Scoping Review and interpretation of the findings. They also supported the development of a plain English summary of project findings that was used as part of a public dissemination event and to increase the reach of study findings for lay audiences and people with life-limiting or life-threatening conditions and their care partners.

## Results

### Selection of sources of evidence

We identified 2393 records on the title and abstract level after removing the duplicates. A total of 34 reports could be included. One of the 34 reports was identified through a hand search by checking the references of the included studies. We received no full texts for 129 reports. The vast majority were conference abstracts for which no full texts were available. The PRISMA 2020 flow chart, as shown in Figure 1, provides an overview of the included and excluded publications and illustrates the reasons for exclusion. A summary of coded extracted data can be found in Table 1, with a comprehensive summary table and definitions of coding in Appendix 2.

### Characteristics of sources of evidence

The first report was published in 2012, and the most recent one is from 2022. A total of 55.8% (19/34) of the reports were published in 2019 or later. All publications appeared in English. Of these, 76.5% (26/34) were from the USA, 14.7% (5/34) were from the UK, 5.9% (2/34) were from Australia, and 2.9% (1/34) were from New Zealand. 47.1% of the reports (*n* = 16/34) were funded by different sources, with 52.9% (18/34) conducted without financial support. Across all studies, 223,550 (range 6–187,690) participants were involved, including patients (*n* = 221,880), healthcare providers (*n* = 399) and ‘Other’ (*n* = 1,271) that included users of online platforms. Most systems were only featured in one report, except for multiple reports relating to MyHealthConnection [20–22], Coordinate My Care [23, 24] and the use of the Epic electronic health record [25–27].

### System characteristics and content (RQ1)

The characteristics and structure of systems described in reports varied. Most systems (*n* = 23;67.7%) were contained within a single EHR system. The remaining systems involved a standalone online platform that was embedded in an EHR (*n* = 6;17.7%), EHRs that enable viewing or editing of content by patients (*n* = 4;11.8%), and ‘other’ (i.e. a mobile phone application-based intervention) (*n* = 1;3.0%).

Reports described systems that enabled recording and sharing of ACP information across different settings. The most common setting with access to systems outlined in reports was the hospital (*n* = 15;44.1%), alongside systems that enabled access to providers across multiple healthcare settings (*n* = 10;29.4%). Systems also included those providing access to providers in outpatient (non-hospital) settings (*n* = 5;14.7%), palliative care providers (*n* = 4;11.8%), preclinical emergency care (*n* = 2;5.9), nursing (*N* = 2;5.9%) and care homes (*n* = 1;2.9%), and ‘other’ care organisations (*n* = 1;2.9%).

Healthcare providers interacted with systems outlined in reports via one of three approaches; access via an EHR system (*n* = 27;79.4%), via a web-based platform designed for healthcare providers that can be accessed by a provider in any care setting (*n* = 6;17.6%), or an online platform enabling professional access to a patient-facing platform where preferences for care are recorded (*n* = 1;2.9%). There was variation in how systems facilitated access between patients and their healthcare providers. These included reports (*n* = 15;44.1%) detailing systems that only enabled healthcare providers’ access to ACP information, either via an EHR system (*n* = 12;35.3%) or a standalone online platform (*n* = 3;8.8%). Around one-third of reports (*n* = 11;32.4%) detailed systems that enabled patients to access and edit records of the ACP information, accessed either via an online platform (*n* = 8;23.5%) or a patient portal linked to an EHR (*n* = 3;8.8%). The content of systems varied across reports, although most systems contained information relating to a patient’s care preferences and healthcare choices (e.g., preferred place of care and death, and advance directives) (*n* = 27;79.4%). Information relating to the identification of advocates and nomination of people to support decision-making (i.e., lasting power of attorney, surrogate decision makers) were present in systems (*n* = 15;44.1%). Also reported to be included in the content of systems were diagnosis and disease status information (*n* = 10;29.4%), personal and social needs (including religious and spiritual preferences) (*n* = 6;17.6%), educational content for patients to support ACP (*n* = 5;14.7%), and medical, nursing, and psychological needs (*n* = 1;2.9%).

### Type of existing studies and assessing outcomes of systems (RQ2)

In 35.3% (12/34) of the reports, a quantitative observational study design was used. Other study designs included qualitative design (*n* = 3;8.8%), quantitative experimental design (*n* = 2;5.9%), and mixed method approaches (*n* = 2;5.9%). The most common study design tended to be ‘other’ non-standard approaches (*n* = 15;44.1%), including quality improvement project reports, commentaries, case reports, and service evaluations.

### Evaluation of systems for electronic documentation and sharing of advance care planning (RQ3)

Across the included studies, multiple types of outcomes were being used to evaluate how systems were supporting and influencing care delivery. The most common type of outcome measures was those focusing on the influence of systems on the documentation of ACP information (*n* = 23;67.6%), particularly relating to the creation, quantity, quality, frequency, or timing of ACP records. Other types of outcome measures included those focusing on the impact of systems of healthcare provider practice (e.g. confidence in undertaking, or number of, ACP conversations) (*n* = 9;26.5%), the impact of systems on health service cost or service utilisation by patients (*n* = 6;17.6%), patient and healthcare providers’ experience and perceptions of the use of a system (*n* = 3;8.8%), satisfaction with documented end-of-life care plans (*n* = 1;2.9%) and the sharing of documentation with surrogates (*n* = 1;2.9%).

## Discussion

### Main findings

This scoping review highlights the characteristics and structure of digital ACP systems currently reported across the international research literature. Most digital ACP approaches were hosted and contained within a single EHR system, either within a single hospital site or intended to facilitate information sharing across multiple settings involved in palliative and end-of-life care delivery. Most digital ACP systems sought to record and share information relating to a patient’s care preferences and healthcare choices, with nearly half able to record an identified advocate or nominate people to support decision-making on behalf of a patient. Included records also reflected the capabilities of systems to support patient access to and editing of their own ACP as recorded on a digital system. The existing evidence reporting digital ACP systems was dominated by US studies, with varied study designs used. Most studies used non-standard designs (e.g. quality improvement project reports, commentaries, and case reports). Where study designs included the assessment of outcomes, these were typically focused on the influence of digital ACP systems on documentation (e.g. quantifying the creation, completeness, frequency, or timing of ACP records). There was a deficit in research exploring outcomes relating to the experience of system users, including both patients and healthcare providers, alongside limited measurement of satisfaction with ACP documented through digital systems.

Digital approaches to the documentation and sharing of patient information and preferences are increasingly being used to support the delivery of high-quality end-of-life care internationally [7–9]. The prominence of non-standard study designs and the recency of records included in the review may reflect the research exploring their impact and implementation as an emerging research area. This research is required to understand how to optimally implement digital ACP systems and crucially to determine if and how they can be useful tools to support the delivery of palliative and end-of-life care [6]. Systems to support healthcare provider documentation and sharing of ACP information in palliative care need to account for the multi-setting and multidisciplinary nature of care delivery [28]. In countries, such as the UK, where systems typically seek to coordinate information sharing across multiple settings, implementation has been fragmented and sub-optimal [29]. For example, most were not able to share information across all providers, often excluding care homes and social care providers from information exchange [29]. Understanding how to optimise the implementation of digital ACP systems is a priority for future research [30].

An emerging capability that was reported in a third of records was the ability of patients to access and edit their own ACP information within EHR systems. This aligns with an increasing recognition of the value of patient access to their records, viewed as an important way of empowering patients in their decision-making related to their health and care [31]. Current content is largely focused on care preferences and healthcare choices, such as place of care and place of death which are viewed as important aspects of care from a patient’s perspective [32]. However, additional factors related to quality of life are known to be important to people receiving palliative care relating to multiple domains including personal autonomy and emotional, social, and spiritual factors [33]. Content relating to these elements was present in fewer than a fifth of the studies included in the review. Patient and carer engagement in the development of digital ACP systems is currently lacking [30] and may be an important next step to explore how well the content of existing ACP systems aligns with aspects of care that are valued by patients. Patient engagement research that accompanies increasing patient access to their record for ACP documentation may also provide opportunities to explore disparities (e.g. those relating to age, disease, and deprivation) that have been identified previously with patient portals for access to medical records [34]. Irrespective of the route developed to facilitate patient access to their own ACP information, research is required to ensure they do not exacerbate known disparities in access to and use of palliative care services [35, 36].

### Strengths and limitations

Although this scoping review provides a detailed insight into the possibilities of digital documentation of ACP preferences, there are some limitations. Firstly, our study was restricted solely to publications written in English. There may be relevant publications from other countries written in the respective language that are not included in our scoping review. Secondly, another limitation is that the primary aim of the included studies was not necessarily to report on the systems used, but mostly to answer other research questions. For example, the most common outcome measure focused on the impact of the systems on the documentation of ACP information. Other types of outcome measures related to the impact of the systems on health providers’ practice (e.g. confidence in conducting ACP conversations). The information relevant to answering the research questions for this scoping review was not always explicitly addressed in the included primary studies. Therefore, misinterpretations cannot be ruled out. However, to avoid misinterpretation as far as possible, the data extraction was carried out by one person and checked by another. Any ambiguities were discussed with another reviewer and the relevant text passages were discussed. Despite these limitations, this scoping review contributes to mapping the available evidence on digital documentation of ACP preferences.

### Implications for practice

Across the research literature, interventions are being reported that may influence ACP before and after the documentation and sharing of APC information using digital systems. These include interventions to increase a person’s willingness and readiness for ACP conversations and decision-making (e.g. [37]). Interventions are also being developed to improve the quality of ACP discussions (e.g. [38]). Following documentation and sharing of ACP documentation, there is an exploration of the review and realisation of care that is concordant with patient wishes (e.g. [39]). Future work may be required across these different elements and emerging interventions to understand any interdependencies. Such research could explore how the different activities influence the engagement of providers and patients with the ACP process and the quality of information that is recorded and shared on digital ACP systems. This may also need to take account of situations in which, irrespective of documentation, challenges exist in providing care in line with documented wishes (e.g. a conflict between honouring preference for comfort care and extending life, changes in patient preferences not being possible to reflect, a lack of resources, and challenges with retrieving ACP documents) [40]. Such research will require the development of more nuanced outcome measures for understanding the impact of digital ACP systems. Within the review, where used, outcomes largely focused on documentation as an endpoint. Future research could seek to understand outcomes relating to, for example, timeliness of access to and use of ACP information accessed via digital systems, or wider implementation outcomes (e.g. appraisal of success in alignment with strategic fit and priority and integration into communications and workflows) [41]. Factors influencing such proximal outcomes relating to the use of systems and their implementation may be a necessary first step before exploring the impact of digital ACP systems on care delivery and patient outcomes.

## Conclusion

Digital ACP approaches are an emerging area for palliative and end-of-life care research, with a predominance of literature from the USA, UK, Australia, and New Zealand. Reported digital ACP approaches use EHR platforms or stand-alone digital platforms, typically supporting documentation in hospitals, nursing homes and outpatient facilities. A fifth of reports detail systems that enable patient access to their records. Future patient and healthcare provider engagement is required to understand the experience of using digital ACP systems and ensure the alignment of system content with what matters to patients. Irrespective of system design, there will be a need to monitor disparities in the use and impact of digital ACP systems and to refine outcome measures to understand their implementation.

## Supplementary Material

Supplemental Material

## Data Availability

The review was conducted using data that were already available in the public domain, with appropriate references provided throughout. The full search strategy used in the present review can be found in the supplementary material.

## References

[1] Payne A. Introducing palliative care, Fourth Edn. Robert Twycross. Radcliffe Medical Press, 2003. 190pp. paperback. ISBN: 1-85775-915-X. Psycho-Oncology 2005;14(1):80–80.

[2] Sudore RL, Lum HD, You JJ, et al. Defining advance care planning for adults: a consensus definition from a multidisciplinary Delphi panel. J Pain Symptom Manage 2017 May;53(5):821–32 e1. 28062339 10.1016/j.jpainsymman.2016.12.331 PMC5728651

[3] Jimenez G, Tan WS, Virk AK, et al. Overview of systematic reviews of advance care planning: summary of evidence and global lessons. J Pain Symptom Manage 2018 Sep;56(3):436–459 e25. 29807158 10.1016/j.jpainsymman.2018.05.016

[4] Detering KM, Hancock AD, Reade MC, Silvester W. The impact of advance care planning on end of life care in elderly patients: randomised controlled trial. BMJ 2010 Mar 23;340:c1345. 20332506 10.1136/bmj.c1345 PMC2844949

[5] Houben CHM, Spruit MA, Groenen MTJ, et al. Efficacy of advance care planning: a systematic review and meta-analysis. J Am Med Dir Assoc 2014 Jul;15(7):477–89. 24598477 10.1016/j.jamda.2014.01.008

[6] Allsop MJ, Chumbley K, Birtwistle J, et al. Building on sand: digital technologies for care coordination and advance care planning. BMJ Support Palliat Care 2022 Jun;12(2):194–7. 10.1136/bmjspcare-2021-003304 34876456

[7] Huber MT, Highland JD, Krishnamoorthi VR, Tang JW. Utilizing the electronic health record to improve advance care planning:a systematic review. Am J Hosp Palliat Care 2018 Mar;35(3):532–41. 28627287 10.1177/1049909117715217

[8] Lamas D, Panariello N, Henrich N, et al. Advance care planning documentation in electronic health records: current challenges and recommendations for change. J Palliat Med 2018 Apr;21(4):522–8. 29360417 10.1089/jpm.2017.0451

[9] McCarthy S, Meredith J, Bryant L, Hemsley B. Legal and ethical issues surrounding advance care directives in Australia: implications for the advance care planning document in the Australian My health record. J Law Med 2017 Nov;25(1):136–49. 29978629

[10] Tricco AC, Tetzlaff J, Moher D. The art and science of knowledge synthesis. J Clin Epidemiol 2011 Jan;64(1):11–20. 20189767 10.1016/j.jclinepi.2009.11.007

[11] Tricco AC, Lillie E, Zarin W, et al. Prisma extension for scoping reviews (PRISMA-ScR): checklist and explanation. Ann Intern Med 2018 Oct 2;169(7):467–73. 30178033 10.7326/M18-0850

[12] (NHS) NHS. End of Life Care Strategy. 2008. Available from: https://assets.publishing.service.gov.uk/government/uploads/system/uploads/attachment_data/file/136431/End_of_life_strategy.pdf.

[13] McGowan J, Sampson M, Salzwedel DM, et al. Press peer review of electronic search strategies: 2015 guideline statement. J Clin Epidemiol 2016 Jul;75:40–6. 27005575 10.1016/j.jclinepi.2016.01.021

[14] Dupont C, Smets T, Monnet F, et al. Publicly available, interactive web-based tools to support advance care planning. Systematic review. J Med Internet Res 2022 Apr 20;24(4):e33320. 35442207 10.2196/33320 PMC9069298

[15] Ouzzani M, Hammady H, Fedorowicz Z, Elmagarmid A. Rayyan-a web and mobile app for systematic reviews. Syst Rev 2016 Dec 5;5(1):210. 27919275 10.1186/s13643-016-0384-4 PMC5139140

[16] Peters MDJ, Godfrey C, McInerney P, Munn Z, Tricco A, Khalil, H. Chapter 11: scoping reviews. In: Aromataris E, Munn Z, editors. JBI manual for evidence synthesis. 2020. Available from: https://synthesismanual.jbi.global

[17] Peters MDJ, Marnie C, Tricco AC, et al. Updated methodological guidance for the conduct of scoping reviews. JBI Evid Synth 2020 Oct;18(10):2119–2126. 33038124 10.11124/JBIES-20-00167

[18] Pollock D, Peters MDJ, Khalil H, et al. Recommendations for the extraction, analysis, and presentation of results in scoping reviews. JBI Evid Synth 2023 Mar 1;21(3):520–32. 36081365 10.11124/JBIES-22-00123

[19] Attride-Stirling J. Thematic networks: an analytic tool for qualitative research. Qual Res 2001;1(3):385–405.

[20] Portz JD, Brungardt A, Shanbhag P, et al. Advance care planning among users of a patient portal during the COVID-19 pandemic: retrospective observational study. J Med Internet Res 2020 Aug 11;22(8):e21385. 32716900 10.2196/21385 PMC7423389

[21] Brungardt A, Daddato AE, Parnes B, Lum HD. Use of an ambulatory patient portal for advance care planning engagement. J Am Board Fam Med 2019 Nov-Dec;32(6):925–30. 31704762 10.3122/jabfm.2019.06.190016 PMC7039311

[22] Jordan SR, Brungardt A, Phimphasone-Brady P, Lum HD. Patient perspectives on advance care planning via a patient portal. Am J Hospice Palliat Care 2019 Aug;36(8):682–7. 10.1177/1049909119832820 PMC6602833 30803245

[23] Riley J, Madill D. Coordinate my care: a clinical approach underpinned by an electronic solution. Progr Palliat Care 2013;21(4):214–9.

[24] Millington-Sanders C, Nadicksbernd JJ, O’Sullivan C, et al. Electronic palliative care co-ordination system: an electronic record that supports communication for end-of-life care - a pilot in Richmond, UK. Lond J Prim Care (Abingdon) 2012;5(1):130–4. 10.1080/17571472.2013.11493399 PMC4413707 25949685

[25] Wilson E, Bernacki R, Lakin JR, et al. Rapid adoption of a serious illness conversation electronic medical record template: lessons learned and future directions. J Palliat Med 2020 Feb;23(2):159–61. 32023189 10.1089/jpm.2019.0420

[26] Wu A, Huang RJ, Colón GR, et al. Low rates of structured advance care planning documentation in electronic health records: results of a single-center observational study. BMC Palliat Care 2022 Nov 22;21(1):203. 36419072 10.1186/s12904-022-01099-9 PMC9686086

[27] Halpert KD, Ward K, Sloane PD. Improving advance care planning documentation using reminders to patients and physicians: a longitudinal study in primary care. Am J Hosp Palliat Care 2022 Jan;39(1):62–7. 33754838 10.1177/10499091211004890

[28] Fernando G, Hughes S. Team approaches in palliative care: a review of the literature. Int J Palliat Nurs 2019 Sep 02;25(9):444–51. 31585054 10.12968/ijpn.2019.25.9.444

[29] Birtwistle J, Millares-Martin P, Evans CJ, et al. Mapping and characterising electronic palliative care coordination systems and their intended impact: A national survey of end-of-life care commissioners. PLoS One 2022;17(10):e0275991. 36240254 10.1371/journal.pone.0275991 PMC9565729

[30] Allsop MJ, Chumbley K, Birtwistle J, et al. Building on sand: digital technologies for care coordination and advance care planning. BMJ Support Palliat Care 2022;12(2):194–7. 10.1136/bmjspcare-2021-003304 34876456

[31] Maria H, Brian M, Robyn W, Charlotte B. Patient empowerment through online access to health records. BMJ 2022;378:e071531. 36175012 10.1136/bmj-2022-071531 PMC9518004

[32] Gomes B, Calanzani N, Gysels M, et al. Heterogeneity and changes in preferences for dying at home: a systematic review. BMC Palliat Care 2013 Feb 15;12:7. 23414145 10.1186/1472-684X-12-7 PMC3623898

[33] McCaffrey N, Bradley S, Ratcliffe J, Currow DC. What aspects of quality of life are important from palliative care patients’ perspectives? A systematic review of qualitative research. J Pain Symptom Manage 2016 Aug;52(2):318–28.e5. 27216362 10.1016/j.jpainsymman.2016.02.012

[34] Grossman LV, Creber M, Benda RM, C N, et al. Interventions to increase patient portal use in vulnerable populations: a systematic review. J Am Med Inform Assoc 2019 Aug 1;26(8–9):855–70. 30958532 10.1093/jamia/ocz023 PMC6696508

[35] French M, Keegan T, Anestis E, Preston N. Exploring socioeconomic inequities in access to palliative and end-of-life care in the UK: a narrative synthesis. BMC Palliat Care 2021 Nov 21;20(1):179. 34802450 10.1186/s12904-021-00878-0 PMC8606060

[36] Koffman J, Shapiro GK, Schulz-Quach C. Enhancing equity and diversity in palliative care clinical practice, research and education. BMC Palliat Care 2023 2023/06/05;22(1):64. 10.1186/s12904-023-01185-6 PMC10239712 37271813

[37] Lum HD, Barnes DE, Katen MT, et al. Improving a full range of advance care planning behavior change and action domains: the PREPARE randomized trial. J Pain Symptom Manage 2018 Oct 01/;56(4):575–81.e7. 29940209 10.1016/j.jpainsymman.2018.06.007 PMC6138565

[38] Hafid A, Howard M, Guenter D, et al. Advance care planning conversations in primary care: a quality improvement project using the serious illness care program. BMC Palliat Care 2021 July 30;20(1):122. 34330245 10.1186/s12904-021-00817-z PMC8325252

[39] Sanders JJ, Curtis JR, Tulsky JA. Achieving goal-concordant care: a conceptual model and approach to measuring serious illness communication and its impact. J Palliat Med 2017 2018 Mar 1;21(S2):S-17–S-27. 10.1089/jpm.2017.0459 PMC5756461 29091522

[40] Malhotra C, Ramakrishnan C, Yue S-MG. Challenges in providing end-of-life care consistent with documented patient preferences. Ann Palliat Med 2022;11(12):3610–3619. 36510456 10.21037/apm-22-790

[41] Wakefield DS, Mehr D, Keplinger L, et al. Issues and questions to consider in implementing secure electronic patient-provider web portal communications systems. Int J Med Inform 2010 Jul;79(7):469–77. 20472495 10.1016/j.ijmedinf.2010.04.005

