# Supplementary material for: Systems for electronic documentation and sharing of advance care planning preferences: a scoping review
Source: Prog Palliat Care. 2024 Apr 26;32(3):149–59. doi: 10.1080/09699260.2024.2339106 (PMC11145469; doi:10.1080/09699260.2024.2339106)
Supplement: Supplemental Material [file YPPC_A_2339106_SM1480.zip › Appendix_1.docx]

**Appendix 1**

**Systems for electronic documentation and sharing of advance care planning preferences: a scoping review**

**Results of literature search**

**Search string, date of search and number of hits:**

[PubMed 1](#_Toc126780595)

[Embase/Elsevier 2](#_Toc126780596)

[CINAHL/EBSCO 2](#_Toc126780597)

[PsycInfo/EBSCO 3](#_Toc126780598)

[ACM Digital Library 4](#_Toc126780599)

[IEEE Explore 5](#_Toc126780600)

[GoogleScholar 6](#_Toc126780601)

[Greylit.org 6](#_Toc126780602)

[Total number of hits 6](#_Toc126780603)

## PubMed

("advance care planning"[MeSH] OR "advance directives"[MeSH] OR "living wills"[MeSH] OR advance care plan*[tiab] OR ACP[tiab] OR pACP[tiab] OR advance decision*[tiab] OR advance directive*[tiab] OR advance medical directive*[tiab] OR advance healthcare planning*[tiab] OR advance medical planning*[tiab] OR advance statement*[tiab] OR "do not hospitalize"[tiab] OR "do not hospitalise"[tiab] OR "do not resuscitate"[tiab] OR "do not attempt cardiopulmonary resuscitation"[tiab] OR "DNR order"[tiab] OR DNACPR[tiab] OR "planning ahead"[tiab] OR "refusal of treatment"[tiab] OR treatment limitation*[tiab])
AND
(applications, medical informatics[MeSH Terms] OR record, electronic health[MeSH Terms] OR record, electronic medical[MeSH Terms] OR health information technology[MeSH Terms] OR design, medical device[MeSH Terms] OR Telemedicine[MeSH Terms] OR m-health[tiab] OR mhealth[tiab] OR e-health[tiab] OR Ehealth[tiab] OR “mobile health”[tiab] OR “mobile phone”[tiab] OR smartphone[tiab] OR “mobile application”[tiab] OR mobile technology[tiab] OR “text message*”[tiab] OR internet[tiab] OR “web-based”[tiab] OR POLST[tiab] OR “Our Care Wishes”[tiab] OR COMPASS[tiab] OR WiserCare[tiab] OR Vynacare[tiab] OR “Living Will Registry”[tiab] OR “Coordinate My Care”[tiab] OR EPaCCS[tiab] OR ePCS[tiab] OR “My Health Record”[tiab] OR ACDCare[tiab] OR “Be my voice”[tiab] OR Beslishulp[tiab] OR Cake[tiab] OR “Dementia Values and Priorities Tool”[tiab] OR “Dying to Talk”[tiab] OR Everplans[tiab] OR “Five Wishes”[tiab] OR “Go Wish card game”[tiab] OR “Lets Think Ahead”[tiab] OR “MyDecisions”[tiab] OR “My living voice”[tiab] OR “my living will”[tiab] OR “My Values”[tiab] OR MyDirectives[tiab] OR MyWishes[tiab] OR NVLivingWill[tiab] OR “Oog in Oog”[tiab] OR “Plan your Life Span”[tiab] OR “Planning for Your Future”[tiab] OR PREPARE[tiab] OR “Speak Up”[tiab] OR “The Letter project”[tiab] OR “Tijdig nadenken over het levenseinde”[tiab] OR “Verken uw wensen voor zorg en behandeling”[tiab]) AND ("2008/01/01"[Date - Publication] : "3000"[Date - Publication])

Date of search: 08.02.2023

Hits: 982

## Embase/Elsevier

('advance care planning'/exp OR 'living will'/exp OR 'advance care plan*':ti,ab OR acp:ti,ab OR pacp:ti,ab OR 'advance decision*':ti,ab OR 'advance directive*':ti,ab OR 'advance medical directive*':ti,ab OR 'advance healthcare planning*':ti,ab OR 'advance medical planning*':ti,ab OR 'advance statement*':ti,ab OR 'do not hospitalize':ti,ab OR 'do not hospitalise':ti,ab OR 'do not resuscitate':ti,ab OR 'do not attempt cardiopulmonary resuscitation':ti,ab OR 'dnr order':ti,ab OR dnacpr:ti,ab OR 'planning ahead':ti,ab OR 'refusal of treatment':ti,ab OR 'treatment limitation*':ti,ab) AND
('medical informatics'/exp OR 'electronic health record'/exp OR 'electronic medical record'/exp OR 'electronic medical record system'/exp OR 'telemedicine'/exp OR 'm-health':ti,ab OR mhealth:ti,ab OR 'e-health':ti,ab OR ehealth:ti,ab OR 'mobile health':ti,ab OR 'mobile phone':ti,ab OR smartphone:ti,ab OR 'mobile application':ti,ab OR 'mobile technology':ti,ab OR 'text message*':ti,ab OR internet:ti,ab OR 'web-based':ti,ab OR polst:ti,ab OR 'our care wishes':ti,ab OR compass:ti,ab OR wisercare:ti,ab OR vynacare:ti,ab OR 'living will registry':ti,ab OR 'coordinate my care':ti,ab OR epaccs:ti,ab OR epcs:ti,ab OR 'my health record':ti,ab OR acdcare:ti,ab OR 'be my voice':ti,ab OR beslishulp:ti,ab OR cake:ti,ab OR 'dementia values and priorities tool':ti,ab OR 'dying to talk':ti,ab OR everplans:ti,ab OR 'five wishes':ti,ab OR 'go wish card game':ti,ab OR 'lets think ahead':ti,ab OR mydecisions:ti,ab OR 'my living voice':ti,ab OR 'my living will':ti,ab OR 'my values':ti,ab OR mydirectives:ti,ab OR mywishes:ti,ab OR nvlivingwill:ti,ab OR 'oog in oog':ti,ab OR 'plan your life span':ti,ab OR 'planning for your future':ti,ab OR prepare:ti,ab OR 'speak up':ti,ab OR 'the letter project':ti,ab OR 'tijdig nadenken over het levenseinde':ti,ab OR 'verken uw wensen voor zorg en behandeling':ti,ab) AND [01-01-2008]/sd NOT [02-09-2023]/sd AND [embase]/lim NOT [medline]/lim NOT [preprint]/lim NOT [pubmed-not-medline]/lim

Date of search: 08.02.2023

Hits: 982

## CINAHL/EBSCO

((MH "advance care planning") OR (MH " advance directives +") OR (MH "living wills") OR (TI "advance care plan*" OR AB "advance care plan*") OR (TI ACP OR AB ACP) OR (TI pACP OR AB pACP) OR (TI "advance decision*" OR AB "advance decision*") OR (TI "advance directive*" OR AB "advance directive*") OR (TI "advance medical directive*" OR AB "advance medical directive*") OR (TI "advance healthcare planning*" OR AB "advance healthcare planning*") OR (TI "advance medical planning*" OR AB "advance medical planning*") OR (TI "advance statement*" OR AB "advance statement*") OR (TI "do not hospitalize" OR AB "do not hospitalize") OR (TI "do not hospitalise" OR AB "do not hospitalise") OR (TI "do not resuscitate" OR AB "do not resuscitate") OR (TI "do not attempt cardiopulmonary resuscitation" OR AB "do not attempt cardiopulmonary resuscitation") OR (TI "DNR order" OR AB "DNR order") OR (TI DNACPR OR AB DNACPR) OR (TI "planning ahead" OR AB "planning ahead") OR (TI "refusal of treatment" OR AB "refusal of treatment") OR (TI "treatment limitation*" OR AB "treatment limitation*"))
AND
((MH "medical informatics") OR (MH "electronic health records+") OR (MH Telemedicine+) OR (TI m-health OR AB m-health) OR (TI mhealth OR AB mhealth) OR (TI e-health OR AB e-health) OR (TI Ehealth OR AB Ehealth) OR (TI "mobile health" OR AB "mobile health") OR (TI "mobile phone" OR AB "mobile phone") OR (TI smartphone OR AB smartphone) OR (TI "mobile application" OR AB "mobile application") OR (TI mobile technology OR AB mobile technology) OR (TI "text message*" OR AB "text message*") OR (TI internet OR AB internet) OR (TI web-based OR AB web-based) OR (TI POLST OR AB POLST) OR (TI "Our Care Wishes" OR AB "Our Care Wishes") OR (TI COMPASS OR AB COMPASS) OR (TI WiserCare OR AB WiserCare) OR (TI Vynacare OR AB Vynacare) OR (TI "Living Will Registry" OR AB "Living Will Registry") OR (TI "Coordinate My Care" OR AB "Coordinate My Care") OR (TI EPaCCS OR AB EPaCCS) OR (TI ePCS OR AB ePCS) OR (TI "My Health Record" OR AB "My Health Record") OR (TI ACDCare OR AB ACDCare) OR (TI "Be my voice" OR AB "Be my voice") OR (TI Beslishulp OR AB Beslishulp) OR (TI Cake OR AB Cake) OR (TI "Dementia Values and Priorities Tool" OR AB "Dementia Values and Priorities Tool") OR (TI "Dying to Talk" OR AB "Dying to Talk") OR (TI Everplans OR AB Everplans) OR (TI "Five Wishes" OR AB "Five Wishes") OR (TI "Go Wish card game" OR AB "Go Wish card game") OR (TI "Lets Think Ahead" OR AB "Lets Think Ahead") OR (TI MyDecisions OR AB MyDecisions) OR (TI "My living voice" OR AB "My living voice") OR (TI "my living will" OR AB "my living will") OR (TI "My Values" OR AB "My Values") OR (TI MyDirectives OR AB MyDirectives) OR (TI MyWishes OR AB MyWishes) OR (TI NVLivingWill OR AB NVLivingWill) OR (TI "Oog in Oog" OR AB "Oog in Oog") OR (TI "Plan your Life Span" OR AB "Plan your Life Span") OR (TI "Planning for Your Future" OR AB "Planning for Your Future") OR (TI PREPARE OR AB PREPARE) OR (TI "Speak Up" OR AB "Speak Up") OR (TI "The Letter project" OR AB "The Letter project") OR (TI "Tijdig nadenken over het levenseinde" OR AB "Tijdig nadenken over het levenseinde") OR (TI "Verken uw wensen voor zorg en behandeling" OR AB "Verken uw wensen voor zorg en behandeling")) AND DT 20080101-20230206

Date of search: 08.02.2023

Hits: 616

## PsycInfo/EBSCO

((DE "advance directives") OR (TI "advance care plan*" OR AB "advance care plan*") OR (TI ACP OR AB ACP) OR (TI pACP OR AB pACP) OR (TI "living will*" OR AB " living will*") OR (TI "advance decision*" OR AB "advance decision*") OR (TI "advance directive*" OR AB "advance directive*") OR (TI "advance medical directive*" OR AB "advance medical directive*") OR (TI "advance healthcare planning*" OR AB "advance healthcare planning*") OR (TI "advance medical planning*" OR AB "advance medical planning*") OR (TI "advance statement*" OR AB "advance statement*") OR (TI "do not hospitalize" OR AB "do not hospitalize") OR (TI "do not hospitalise" OR AB "do not hospitalise") OR (TI "do not resuscitate" OR AB "do not resuscitate") OR (TI "do not attempt cardiopulmonary resuscitation" OR AB "do not attempt cardiopulmonary resuscitation") OR (TI "DNR order" OR AB "DNR order") OR (TI DNACPR OR AB DNACPR) OR (TI "planning ahead" OR AB "planning ahead") OR (TI "refusal of treatment" OR AB "refusal of treatment") OR (TI "treatment limitation*" OR AB "treatment limitation*"))
AND
((DE "mobile applications+") OR (DE "electronic health records+") OR (DE Telemedicine+) OR (TI m-health OR AB m-health) OR (TI mhealth OR AB mhealth) OR (TI e-health OR AB e-health) OR (TI Ehealth OR AB Ehealth) OR (TI "mobile health" OR AB "mobile health") OR (TI "mobile phone" OR AB "mobile phone") OR (TI smartphone OR AB smartphone) OR (TI "mobile application" OR AB "mobile application") OR (TI mobile technology OR AB mobile technology) OR (TI "text message*" OR AB "text message*") OR (TI internet OR AB internet) OR (TI web-based OR AB web-based) OR (TI POLST OR AB POLST) OR (TI "Our Care Wishes" OR AB "Our Care Wishes") OR (TI COMPASS OR AB COMPASS) OR (TI WiserCare OR AB WiserCare) OR (TI Vynacare OR AB Vynacare) OR (TI "Living Will Registry" OR AB "Living Will Registry") OR (TI "Coordinate My Care" OR AB "Coordinate My Care") OR (TI EPaCCS OR AB EPaCCS) OR (TI ePCS OR AB ePCS) OR (TI "My Health Record" OR AB "My Health Record") OR (TI ACDCare OR AB ACDCare) OR (TI "Be my voice" OR AB "Be my voice") OR (TI Beslishulp OR AB Beslishulp) OR (TI Cake OR AB Cake) OR (TI "Dementia Values and Priorities Tool" OR AB "Dementia Values and Priorities Tool") OR (TI "Dying to Talk" OR AB "Dying to Talk") OR (TI Everplans OR AB Everplans) OR (TI "Five Wishes" OR AB "Five Wishes") OR (TI "Go Wish card game" OR AB "Go Wish card game") OR (TI "Lets Think Ahead" OR AB "Lets Think Ahead") OR (TI MyDecisions OR AB MyDecisions) OR (TI "My living voice" OR AB "My living voice") OR (TI "my living will" OR AB "my living will") OR (TI "My Values" OR AB "My Values") OR (TI MyDirectives OR AB MyDirectives) OR (TI MyWishes OR AB MyWishes) OR (TI NVLivingWill OR AB NVLivingWill) OR (TI "Oog in Oog" OR AB "Oog in Oog") OR (TI "Plan your Life Span" OR AB "Plan your Life Span") OR (TI "Planning for Your Future" OR AB "Planning for Your Future") OR (TI PREPARE OR AB PREPARE) OR (TI "Speak Up" OR AB "Speak Up") OR (TI "The Letter project" OR AB "The Letter project") OR (TI "Tijdig nadenken over het levenseinde" OR AB "Tijdig nadenken over het levenseinde") OR (TI "Verken uw wensen voor zorg en behandeling" OR AB "Verken uw wensen voor zorg en behandeling")) AND DT 20080101-20230206

Date of search: 08.02.2023

Hits: 180

## ACM Digital Library

**Searched The ACM Guide to Computing Literature**

(Title:("advance care plan*" acp OR pacp "living will*" "advance decision*" "advance directive*" "advance medical directive*" "advance healthcare planning*" "advance medical planning*" "advance statement*" "do not hospitalize" "do not hospitalise" "do not resuscitate" "do not attempt cardiopulmonary resuscitation" "dnr order" dnacpr "planning ahead" "refusal of treatment" "treatment limitation*") AND Title:("electronic health record*" "electronic medical record*" "health information technolog*" Telemedicine OR m\-health OR mhealth OR e\-health OR Ehealth "mobile health" "mobile phone" smartphone "mobile application" "mobile technology" "text message*" internet OR "web\-based" OR POLST "Our Care Wishes" COMPASS OR WiserCare OR Vynacare "Living Will Registry" "Coordinate My Care" EPaCCS OR ePCS "My Health Record" ACDCare "Be my voice" OR Beslishulp Cake "Dementia Values and Priorities Tool" "Dying to Talk" Everplans "Five Wishes" "Go Wish card game" "Lets Think Ahead" "MyDecisions" "My living voice" "my living will" "My Values" MyDirectives OR MyWishes OR NVLivingWill "Oog in Oog" "Plan your Life Span" "Planning for Your Future" PREPARE "Speak Up" "The Letter project" "Tijdig nadenken over het levenseinde" "Verken uw wensen voor zorg en behandeling"))
OR
(Abstract:("advance care plan*" acp OR pacp "living will*" "advance decision*" "advance directive*" "advance medical directive*" "advance healthcare planning*" "advance medical planning*" "advance statement*" "do not hospitalize" "do not hospitalise" "do not resuscitate" "do not attempt cardiopulmonary resuscitation" "dnr order" dnacpr "planning ahead" "refusal of treatment" "treatment limitation*") AND Abstract:("electronic health record*" "electronic medical record*" "health information technolog*" Telemedicine OR m\-health OR mhealth OR e\-health OR Ehealth "mobile health" "mobile phone" smartphone "mobile application" "mobile technology" "text message*" internet OR "web\-based" OR POLST "Our Care Wishes" COMPASS OR WiserCare OR Vynacare "Living Will Registry" "Coordinate My Care" EPaCCS OR ePCS "My Health Record" ACDCare "Be my voice" OR Beslishulp Cake "Dementia Values and Priorities Tool" "Dying to Talk" Everplans "Five Wishes" "Go Wish card game" "Lets Think Ahead" "MyDecisions" "My living voice" "my living will" "My Values" MyDirectives OR MyWishes OR NVLivingWill "Oog in Oog" "Plan your Life Span" "Planning for Your Future" PREPARE "Speak Up" "The Letter project" "Tijdig nadenken over het levenseinde" "Verken uw wensen voor zorg en behandeling"))
E-Publication Date: (01/01/2008 TO 02/28/2023)

Date of search: 08.02.2023

Hits:73

## IEEE Explore

("Index Terms":"advance care planning" OR "Index Terms":"advance directive*" OR "Index Terms":"living wills" OR "Document Title":"advance care plan*" OR "Abstract":"advance care plan*" OR "Document Title":ACP OR "Abstract":ACP OR "Document Title":pACP OR "Abstract":pACP OR "Document Title":"advance decision" OR "Abstract":"advance decision" OR "Document Title":"advance medical directives" OR "Abstract":"advance medical directives" OR "Document Title":"advance healthcare planning" OR "Abstract":"advance healthcare planning" OR "Document Title":"advance medical planning" OR "Abstract":"advance medical planning" OR "Document Title":"advance statement*" OR "Abstract":"advance statement*" OR "Document Title":"do not hospitalize" OR "Abstract":"do not hospitalize" OR "Document Title":"do not hospitalise" OR "Abstract":"do not hospitalise" OR "Document Title":"do not resuscitate" OR "Abstract":"do not resuscitate" OR "Document Title":"do not attempt cardiopulmonary resuscitation" OR "Abstract":"do not attempt cardiopulmonary resuscitation" OR "Document Title":"DNR order" OR "Abstract":"DNR order" OR "Document Title":DNACPR OR "Abstract":DNACPR OR "Document Title":"planning ahead" OR "Abstract":"planning ahead" OR "Document Title":"refusal of treatment" OR "Abstract":"refusal of treatment" OR "Document Title":"treatment limitation*" OR "Abstract":"treatment limitation*")
AND
("Index Terms":"applications, medical informatics" OR "Index Terms":"electronic health records" OR "Index Terms":"electronic medical record" OR "Index Terms":"health information technology" OR "Index Terms":"medical device design" OR "Index Terms":"Telemedicine" OR "Document Title":m-health OR "Abstract":m-health OR "Document Title":mhealth OR "Abstract":mhealth OR "Document Title":e-health OR "Abstract":e-health OR "Document Title":"mobile health" OR "Abstract":"mobile health" OR "Document Title":"mobile phone" OR "Abstract":"mobile phone" OR "Document Title":smartphone OR "Abstract":smartphone OR "Document Title":"mobile application" OR "Abstract":"mobile application" OR "Document Title":mobile technology OR "Abstract":mobile technology OR "Document Title": "text messages" OR "Abstract": "text messages" "Document Title":internet OR "Abstract":internet OR "Document Title":"web-based" OR "Abstract":"web-based" OR "Document Title":POLST OR "Abstract":POLST OR "Document Title":"Our Care Wishes" OR "Abstract":"Our Care Wishes" OR "Document Title":COMPASS OR "Abstract":COMPASS OR "Document Title":WiserCare OR "Abstract":WiserCare OR "Document Title":Vynacare OR "Abstract":Vynacare OR "Document Title":"Living Will Registry" OR "Abstract":"Living Will Registry OR "Document Title":"Coordinate My Care" OR "Abstract":"Coordinate My Care" OR "Document Title":EPaCCS OR "Abstract":EPaCCS OR "Document Title":ePCS OR "Abstract":ePCS OR "Document Title":"My Health Record" OR "Abstract":"My Health Record" OR "Document Title":ACDCare OR "Abstract":ACDCare OR "Document Title":"Be my voice" OR "Abstract":"Be my voice" OR "Document Title":Beslishulp OR "Abstract":Beslishulp OR "Document Title":Cake OR "Abstract":Cake OR "Document Title":"Dementia Values and Priorities Tool" OR "Abstract":"Dementia Values and Priorities Tool" OR "Document Title":"Dying to Talk" OR "Abstract":"Dying to Talk" OR "Document Title":Everplans OR "Abstract":Everplans OR "Document Title":"Five Wishes" OR "Abstract":"Five Wishes" OR "Document Title":"Go Wish card game" OR "Abstract":"Go Wish card game" OR "Document Title":"Lets Think Ahead" OR "Abstract":"Lets Think Ahead" OR "Document Title":"MyDecisions" OR "Abstract":"MyDecisions" OR "Document Title":"My living voice" OR "Abstract":"My living voice" OR "Document Title":"my living will" OR "Abstract":"my living will" OR "Document Title":"My Values" OR "Abstract":"My Values" OR "Document Title":MyDirectives OR "Abstract":MyDirectives OR "Document Title":MyWishes OR "Abstract":MyWishes OR "Document Title":NVLivingWill OR "Abstract":NVLivingWill OR "Document Title":"Oog in Oog" OR "Abstract":"Oog in Oog" OR "Document Title":"Plan your Life Span" OR "Abstract":"Plan your Life Span" OR "Document Title":"Planning for Your Future" OR "Abstract":"Planning for Your Future" OR "Document Title":PREPARE OR "Abstract":PREPARE OR "Document Title":"Speak Up" OR "Abstract":"Speak Up" OR "Document Title":"The Letter project" OR "Abstract":"The Letter project" OR "Document Title":"Tijdig nadenken over het levenseinde" OR "Abstract":"Tijdig nadenken over het levenseinde" OR "Document Title":"Verken uw wensen voor zorg en behandeling" OR "Abstract":"Verken uw wensen voor zorg en behandeling")
Filter applied: 2008-2023

Date of search: 08.02.2023

Hits: 32

## GoogleScholar

allintitle:("advance directives|decisions|statements"|"advance care planning"|"do not resuscitate")("electronic health|medical records"|"telemedicine"|"m|e health"|mhealth|ehealth|"mobile health|phone|application|technology"|smartphone|internet|web-based)
Filter applied: 2008-2023

Date of search: 08.02.2023

Hits: 34

## Greylit.org

"advance care" AND electronic

Date of search: 08.02.2023

Hits: 5

## Total number of hits

Before deduplication: 2904

After deduplication ^[[1]](#footnote-1)^: 2392

1. Deduplication was conducted using *Deduklick*, an AI-based deduplication algorithm:

   [Borissov N, Haas Q, Minder B, Kopp-Heim D, von Gernler M, Janka H, Teodoro D, Amini P. Reducing systematic review burden using Deduklick: a novel, automated, reliable, and explainable deduplication algorithm to foster medical research. Syst Rev. 2022 Aug 17;11(1):172. doi: 10.1186/s13643-022-02045-9. PMID: 35978441; PMCID: PMC9382798](https://www.ncbi.nlm.nih.gov/pmc/articles/PMC9382798/). [↑](#footnote-ref-1)
