# Supplementary material for: Systems for electronic documentation and sharing of advance care planning preferences: a scoping review
Source: Prog Palliat Care. 2024 Apr 26;32(3):149–59. doi: 10.1080/09699260.2024.2339106 (PMC11145469; doi:10.1080/09699260.2024.2339106)
Supplement: Supplemental Material [file YPPC_A_2339106_SM1480.zip › Appendix_2.docx]

**Appendix 2: Summary of coded categorisation of included reports including definitions of categories.**

| Author(s) | Year | Country | Name / title of system being described | Aim | Study design | Population and Sample size (n) | Description of system(s) involved | Settings | Description about the ways that different health professionals interact with the system | Processes for interacting with the system (health professional and/or patient) or receiving communication from the system | Content of the system (i.e. details of types of data recorded by system) | Outcomes evaluated (study outcomes) |
| --- | --- | --- | --- | --- | --- | --- | --- | --- | --- | --- | --- | --- |
| Reidy et al | 2017 | USA | Luminat | Describe early experiences in building a systematic, population-based ACP initiative focused on health system-wide deployment of an Internet-based tool as an adjunct to a facilitator-based model | e | N=27 (hospitalists, residents, and nurse practitioners on conducting goals-of-care conversations - although other survey work also outlined) | a | f | a | b | f | a, c |
| Rolnick et al | 2021 | USA | Our Care Wishes | To compare a web-based, EHR-transmissible AD to a paper AD | b | N=91 (Adults with gastrointestinal and lung malignancies were recruited from the Penn Medicine Infusion Center. 46 randomly allocated to the web AD and 45 to paper) | c | c | a, b | d | b | a, d, e |
| Saiki et al | 2017 | USA | Goals of Care template, as part of TEAM approach (Time, Education, Assessment, and Management) | Provide overview of existing goals of care template within an electronic medical record | e | NA | a | c | a | g | a, b, c, d, e | N/A |
| Seecof et al | 2022 | USA | NA | To provide an update to a prior study from our institution that outlined the need for increased documentation of advance care planning (ACP) in an urban geriatric population. | e | N=50 (Patients at the Center for Healthy Aging aged 65 or older without documented ACP in the EMR) | a | g | a | e | a, b, c, e | c |
| Serrano-Eanelli et al | 2019 | USA | New York State eMOLST | To investigate whether signing of eMOLST results in any reduction in length of stay and direct costs for a community-based hospital in New York State. | e | N=331 (medical records of patients) | c | h | a | a, c | a, b | b |
| Stepan et al | 2019 | USA | NA | To identify needed resources, develop an intervention to improve ACP, and evaluate the intervention’s effects. | e | N=~15,600 (patient records of all new patients with a diagnosis of advanced or metastatic cancer who had a documented advance directive and/or ACP conversations with an ambulatory care provider (physicians, fellows, residents, physician assistants, nurse practitioners, nurses, social work counselors, and chap- lains) by their third office visit) | a | h | a | e | a, b, c | c |
| Tieu et al | 2017 | USA | Mayo Clinic Patient Online Services system | Examine whether the use of ACP-specific patient electronic messages would increase rates of AD completion in patients aged 65 years and older in an academic primary care practice | c | N=200 (patients within the Division of Primary Care Internal Medicine, aged 65 and older, with access to the secure PEM system and without an AD on file, were included for randomization) | c | c | a | b | b, c, e | a, b |
| Walker et al | 2018 | USA | Prepare for your care | To describe ACP documentation practices and the accessibility of documented discussions in the electronic health record | c | N=414 (primary care patients at the San Francisco Veterans Affairs Medical Center, were $60 years old, and had $2 chronic/serious health conditions) | a | h | a | c | b, c | a |
| Wilson et al | 2020 | USA | Epic Systems EMR | To report on an EMR template that captures the dynamic process of ACP yet minimizes cognitive overload, enabling clinicians to find information. | e | NA | a | h | a | e | a, c, e | a, c |
| Wu et al | 2022 | USA | Epic Systems | Evaluate de-identified data derived from outpatient clinics at a single institution and examined rates of Structured ACP documentation at the clinic level.  Also sought to understand clinic- and provider-level factors which are related to Structured ACP completion. | c | N=187,690 (unique patients 65 years and older with at least one clinical encounter at an SHC outpatient clinic over the study period) | a | h | a | a | a, b, c | a |
| Wye et al | 2016 | UK | ADASTRA (UK EHR) | To understand this relationship between EPaCCS and home death better | d | N=3,594 (Data from 1,022 North Somerset and 2,572 Somerset palliative patients; interview participants = 101 professionals, 49 by telephone, 29 face to face and 23 informally) | a | h | a | a | a, b | a, b |
| Zive et al | 2016 | USA | ePOLST | To highlight the technical development and pilot testing process of an ePOLST system, and describe data about use and error rates during early implementation | e | NA. Intended for those nearing end of life. | a | c | a | b | b, c, f | N/A |
| Shervin Esfahani et al | 2020 | USA | SmartPhrase (a tool in the Epic EMR system) | To assess the frequency and quality of goals-of-care conversation documentation among patients who died in an intensive care unit.  To develop and implement mechanisms to improve the quantity and quality of goals-of-care conversations. | e | N=365 (Retrospective record review of all ICU deaths across the system in 1 year) | a | h | a | e | a, b, c | a |
| Goodwin et al | 2021 | New Zealand | eAC Plans | To describe the development of an ACP service and discuss its impact and challenges encountered during its introduction | e | N=3,238 (The findings were obtained from an analysis of a database of 3238 people who had finalised an AC Plan between December 2013 and December 2019.) | a | a, c, e | a | e | b | a, c |
| Hall et al | 2012 | UK (Scotland) | Electronic Palliative Care Summaries (ePCSs) | To identify key issues related to the introduction of ePCS from primary care and OOH staff, to identify facilitators and barriers to their use, to explore the experiences of patients and carers and to make recommendations for improvements. | a | N=22 (purposive sampling of practice nurses (3 interviews), GPs (12 interviews), a practice manager (1 interview) from practices using different computing software systems] and patients and/or carers (6 interviews for whom an ePCS had been completed)) | a | a, b, c. | a | e | a, b | c |
| Halpert et al | 2021 | USA | EPIC EMR, which includes an ACP activity tab | To examine the use of patient and provider reminders to trigger advance care planning discussions in a primary care practice. | c | N=426 (patients aged 75 years or older who had an upcoming appointment with their provider within 2 weeks and who had no documentation of ACP in their chart) | a | c | a | g | b, c | a, b |
| Harrington et al | 2020 | UK (England) | NA | To review and improve practice in relation to DNACPR documentation and communication on gerontology wards in the trust. | d | N=159 (data on gerontology inpatients (n=133) with electronic do not attempt cardiopulmonary resuscitation (e-DNACPR) decisions and survey data from staff (n=26) including senior and junior doctors, and medical trainees) | a | c | a | e | b, f | a, c |
| Portz et al | 2020 | USA | My Health Connection | To determine the rates of use of a web-based advance care planning tool through a health system–based electronic patient portal both before and in the early months of the COVID-19 pandemic. | c | N=3292 (UCHealth patients who interacted with the My Health Connection patient portal, advance care planning tools, specifically by completing an electronic MDPOA form or sending an electronic message to the advance care planning support team) | a | c | a | d | c, f | a |
| Portz et al | 2020 | USA | My Health Manager (Kaiser Permanente Colorado’s (KPCO) patient portal) | To understand the perceptions of older patients with multiple chronic conditions about using the patient portal for ACP and AD documentation | a | N=24 (participants from the KPCO EMR with inclusion criteria included: ≥65 years of age, KPCO member for ≥ 1 year, **presence of multiple chronic conditions (**Charlson Comorbidity Index > 2), and a patient with one of the participating KPCO study clinics) | a | c | a | b | b | a |
| Riley et al | 2013 | UK (England) | Coordinate My Care | To provide an overview of digital advance care planning system that has been used in London, UK | e | NA | b | a, b, c, d, e | b | f | b | NA |
| Auret et al | 2019 | Australia | NA | To create a system for storing, accessing and incorporating ACP documents in clinical care | e | N=73 (Physicians (n=26), nurses (n=15) other HCPs (n=12), audit of patients (n=20)) | a | c | a | e | b, c | a, c |
| Seuli Bose-Brill et al | 2016 | USA | MyChart | To test a novel personal health record (PHR)-delivered ACP framework through a small-scale randomized trial of usual care practices versus PHR-delivered ACP | b | N=50 (patients) | c | a | a | b | b | a |
| Brungardt et al | 2019 | USA | My Health Connection | To increase ACP outcomes by engaging older adults through portal-based ACP tools, in- cluding an electronic Medical Durable Power of Attorney (MDPOA) form | c | N=105 (patients) | a | a | a | d | c | a, c |
| Holt et al | 2019 | USA | Registry of DNR orders | Tattoos and medallions are examples of nonstandard do-not-resuscitate (DNR) orders that some people use to convey end-of-life wishes. These DNR orders are neither universally accepted nor understood for reasons discussed within this manuscript. | e | NA | b | h | b | f | b | N/A |
| Jordan et al | 2019 | USA | My Health Connection | To describe patient perspectives on use of patient portal-based ACP tools. | a | N=46 (patients, mean age: 49, 63% female) | a | h | a | b | c, f | a |
| Klugman et al | 2013 | USA | TexasLivingWill.org and NVLivingWill.com | The aim is to evaluate the terms of use of the tool. Questions are asked about experiences with the use and reasons for filling out an advance directive and about the content. In addition, technical aspects are asked about and where he or she keeps the document and with whom he or she has discussed the wishes at the end of life. with whom he or she discussed the wishes at the end of life. | c | N=371 (platform users) | b | NA | b | b | b, c | h |
| McDarby et al | 2021 | USA | ACP Tools (iOS and Android) BIDMC Health Care ContingencyPlan— Personal  MedStar CR My Dot Mediq  My Directives My Health Proxy My Living Will Paper Health | This review identifies limitations in features, design quality, and content of existing advance care planning mobile apps | e | NA | d | NA | NA | NA | NA | h |
| Millington-Sanders et al | 2012 | UK | Coordinate My Care (CMC) | The aim is to outline the effects of the EPaCCs in Richmond, in respect of (1) enabling patient choice over place of death, and (2) reducing hospital costs. | c | N=597 (patients) | b | h | b | f | b | a, b |
| Mills et al | 2021 | Australia | MyHealth Record | The aim is to explore palliative care practitioners’ current use of and perspectives on digital health innovation in palliative care. | e | N=170 (Medical, nursing, and allied health practitioners working in palliative care) | b | g | a, b | f | f | NA |
| Moses et al | 2020 | USA | NA | The aim is to provide tips for overcoming common barriers to implementing ACP initiatives in an EHR-driven world. | e | NA | a | NA | NA | NA | NA | a,b,f |
| Nakagawa et al | 2014 | USA | NA | The aim is to to examine the quality of ACP documentation after the implementation of a new Veterans Affairs (VA) EMR ACP template. | c | N=93 (patients) | a | c | a | e | b, c, e | a |
| Neubauer et al | 2015 | USA | My Choices, My Wishes | The aim is to investigate the frequency of documentation of coding status in outpatient oncology. | c | N=5,467 (patients) | a | c | a | e | b | a |
| Obel et al | 2014 | USA | NA | The project included creation of new workflow; development of an ACP patient education guidebook; training seminars for oncology staff; and enhancements to the electronic health record (EHR) to improve ACP documentation. | c | N=48 (patients) | a | c | a | e | a, b | a |
| Fin et al | 2016 | USA | MyDirectives | To determine the results of a digital ACP/AD through which consumers create, store, locate, and retrieve their ACP/AD at no charge and with minimal physician involvement, and the ACP/AD can be integrated into the electronic health record | c | N=900 (people who were users of the MyDirectives platform) | b | h | c | b | b, e | a |

Overview of the coding system used for categorising elements of included records, including definitions of each category.

| **Category** | **Code** | **Definition** |
| --- | --- | --- |
| ***Study design*** | 1. Qualitative | Qualitative study design |
|  | 1. Quantitative (experimental) | Quantitative (experimental) study design |
|  | 1. Quantitative (observational) | Quantitative (observational) study design |
|  | 1. Mixed | Mixed method approach |
|  | 1. other | Not categorised within one of above categories |
| ***Description of system(s) involved*** | 1. Electronic Health Record (EHR) | A system providing a digital version of a patient’s paper chart and enabling the exchange of patient data among healthcare providers and institutions, such as laboratories, specialists, medical imaging facilities, pharmacies, emergency facilities, and school and workplace clinics – so they contain information from all clinicians involved in a patient’s care. |
|  | 1. Standalone system not involving an EHR | A stand-alone system, in contrast to an EHR, that does not exchange data with an EHR or other electronic health information systems. Generally, it can be used for specific tasks or functions within a healthcare facility but does not contribute to the comprehensive patient record stored in an EHR. Here, it is to be understood as a system in which documentation relating to palliative and end-of-life care can be stored. |
|  | 1. Standalone system not involving an EHR | Digital tools and platforms designed to facilitate the process of recording, storing, and sharing an individual's preferences and decisions regarding their medical care and treatment choices at the end of life (e.g., **recording and documenting preferences, storing and managing information, sharing with healthcare providers, sharing with family and caregivers, and providing information to guide advance care planning decision making).** |
|  | 1. d) EHR Plus | Includes additional functions beyond the standard EHR to support enhanced documentation and management of patient's end-of-life wishes. |
|  | 1. Other | Systems that cannot be included in one of the existing categories above. |
| ***Settings*** | 1. Outpatient (not hospital), primary care and community teams | This category encompasses healthcare services and teams that provide medical care to patients outside of a hospital setting. It includes primary care practices, community health centres, and outpatient clinics that offer a wide range of non-hospital-based medical services. |
|  | 1. Preclinical emergency care (e.g. ambulance providers) | This category involves the initial emergency medical care provided to individuals before they reach a hospital or clinical facility. It involves ambulance providers, paramedics who deliver urgent medical care and transportation to patients experiencing emergencies. |
|  | 1. Hospital settings | Hospital settings refer to the physical facilities and medical environments where inpatient care is provided. This includes general hospitals, specialized hospitals, and other healthcare facilities designed to provide comprehensive medical treatment, surgeries, and specialized care. |
|  | 1. Care home | A care home, is a residential institution where individuals, often elderly or those with specific medical needs, receive assistance and care with daily living activities, including medical care and support for chronic conditions. |
|  | 1. Nursing homes | Nursing homes are a subset of care homes, primarily focused on providing long-term care for individuals who require assistance with activities of daily living and medical care. They are staffed with trained healthcare professionals, including nurses, to cater to the needs of residents. |
|  | 1. Care Organization | This category refers to a structured healthcare entity or organization responsible for providing care, support, and services to patients or clients. It includes a range of healthcare providers, facilities, and services, working together to deliver comprehensive care. |
|  | 1. Palliative care | Palliative care is a specialized form of healthcare focused on providing comfort, symptom management, and emotional support to individuals with serious illnesses, especially those who are nearing the end of life. The goal is to improve the quality of life for patients and their families. |
|  | 1. Across multiple settings | This category indicates that care, treatment, or services are delivered to patients across various healthcare settings. It may involve a coordinated approach to healthcare that spans outpatient, hospital, home care, and other settings as needed. |
|  | Not applicable (NA) | The category is used if the above categories do not apply. |
| ***Description of the ways in which different health professionals interact with the system*** | 1. Access via EHR (Electronic Health Record) | Health professionals can access the system through an integrated Electronic Health Record (EHR) system. This means that the system is seamlessly connected to the patient's electronic health record, allowing authorized healthcare providers to view and update relevant patient information directly within the EHR. |
|  | 1. Web-based and can be accessed by any legitimate provider of care | The system is web-based, which means it is accessible via a secure online platform. It can be accessed by any legitimate healthcare provider, such as physicians, nurses, and specialists, who have the necessary credentials and permissions. |
|  | 1. Standalone system for electronic recording of ACP Plans | This description refers to a system that is designed as a dedicated and independent electronic platform for recording and managing ACP. It is not integrated into a larger EHR but serves the specific purpose of documenting and storing ACP documentation electronically. |
|  | Not applicable (NA) | The category is used if the above categories do not apply. |
| ***Processes for interacting with the system (health professional and/or patient) or receiving*** | 1. **Patient access via EHR (view only)** | Patients have the capability to access their own electronic health records (EHR) but can only view the information. They do not have the permission to make changes or edits to the content |
|  | 1. **Patient access via application (editing possible)** | Patients can access the system through a dedicated application, which allows them to view and, importantly, edit or update their own health information and care plans. |
|  | 1. **Patient access via patient portal/website (view only)** | Patients have access to their healthcare information through a secure patient portal or website, but their access is limited to viewing the information, without the ability to make changes. |
|  | 1. **Patient access via patient portal (editing possible)** | Patients can access their healthcare information through a patient portal or website, but they are granted the ability to edit or update their own health records and care plans. |
|  | 1. **Patient access via patient portal (editing possible)** | Patients can access their healthcare information through a patient portal or website, but they are granted the ability to edit or update their own health records and care plans. |
|  | 1. **Mobile connectivity for capturing real-time signatures** | The system allows for the real-time capture of electronic signatures, which can include signatures from both healthcare professionals involved in completing forms and patients or their designated surrogates who are present during healthcare discussions. |
|  | 1. **Patient access via printed copy** | Patients are provided with a printed copy of relevant healthcare information, forms, or care plans. They may receive a physical document that contains their medical details and preferences for their records |
|  | Not applicable (NA) | The category is used if the above categories do not apply. |
| ***Content of the system (i.e. details of types of data recorded by system)*** | 1. **Diagnosis / disease status** | **Diagnosis / disease status can be recorded by the system** |
|  | 1. **Care preferences/health care choices (e.g. PPD), advance directives)** | **Care preferences/health care choices (e.g. PPD), advance directives) can be recorded by the system** |
|  | 1. **Lasting power of attorney, surrogate decision maker** | **Lasting power of attorney, surrogate decision maker can be recorded by the system** |
|  | 1. **medical, nursing, psychological needs** | **medical, nursing, psychological needs can be recorded by the system** |
|  | 1. **Patient story/persona/social needs and values:** | **Patient story/persona/social needs and values: (e.g. religious and spiritual preferences) can be recorded by the system** |
|  | 1. **Educational content** | **Educational content can be recorded by the system** |
| ***Outcomes measured and reported in included reports*** | **a) Influence on the creation, quantity, quality, frequency or timing of documentation of ACP in EHR including presence or absence of a health-care proxy** | **Influence on the creation, quantity, quality, frequency or timing of documentation of ACP in EHR including presence or absence of a health-care proxy measured and reported** |
|  | **b) Impact on health service cost and/or utilisation** | **Impact on health service cost and/or utilisation measured and reported** |
|  | **c) Impact on health professional practice (e.g., confidence, number of ACP conversations)** | **Impact on health professional practice (e.g. confidence, number of ACP conversations) measured and reported** |
|  | **d) Satisfaction with end-of-life plans and systems** | **Satisfaction with end-of-life plans and systems measured and reported** |
|  | **e) Sharing documentation with surrogates** | **Sharing documentation with surrogates measured and reported** |
|  | **f) Patient/health provider experience and perceptions of platform** | **Patient/health provider experience and perceptions of platform measured and reported** |
